# Tau Protein and Adult Hippocampal Neurogenesis

**DOI:** 10.3389/fnins.2012.00104

**Published:** 2012-07-09

**Authors:** Almudena Fuster-Matanzo, María Llorens-Martín, Jerónimo Jurado-Arjona, Jesús Avila, Félix Hernández

**Affiliations:** ^1^Centro de Biología Molecular Severo Ochoa, Consejo Superior de Investigaciones Científicas-Universidad Autónoma de Madrid, (CSIC/UAM)Madrid, Spain; ^2^CIBERNED, Centro de Investigación Biomédica en Red de Enfermedades NeurodegenerativasMadrid, Spain

**Keywords:** adult neurogenesis, hippocampus, phosphorylation, splicing, tau

## Abstract

Tau protein is a microtubule-associated protein found in the axonal compartment that stabilizes neuronal microtubules under normal physiological conditions. Tau metabolism has attracted much attention because of its role in neurodegenerative disorders called tauopathies, mainly Alzheimer disease. Here, we review recent findings suggesting that axonal outgrowth in subgranular zone during adult hippocampal neurogenesis requires a dynamic microtubule network and tau protein facilitates to maintain that dynamic cytoskeleton. Those functions are carried out in part by tau isoform with only three microtubule-binding domains (without exon 10) and by presence of hyperphosphorylated tau forms. Thus, tau is a good marker and a valuable tool to study new axons in adult neurogenesis.

## Introduction

The subgranular zone (SGZ) of the hippocampal dentate gyrus is one of the regions where adult neurogenesis takes place. Newborn granule cells generated in the SGZ grow dendrites into the molecular layer and send axons into the CA3 region. This process is similar to that which occurs during neuronal polarization mainly studied with rodent embryonic hippocampal neurons *in vitro* (Dotti et al., 1988, for a review see Kaech and Banker, 2006). Neuronal polarity is mainly due to a polarized cytoskeleton and polarized distribution of cytoskeleton-associated molecules. One of those axonal proteins is tau protein. This review focuses on recent data showing the importance of tau protein in adult neurogenesis. Thus, outgrowth of axons during embryonic neurogenesis is characterized by the expression of tau isoforms with less affinity by the microtubules, tau with three microtubule-binding domains (tau-3R). In addition, tau protein is found in these new neurons in a hyperphosphorylated form. These findings suggest that axonal outgrowth requires a dynamic microtubule network and tau protein facilitates to maintain that dynamic cytoskeleton.

## Hippocampal Adult Neurogenesis

New neurons in the SGZ of the hippocampal formation grow dendrites into the molecular layer, and send axons into the CA3 region. Major glutamatergic synaptic activation from perforant path afferents does not occur until new neurons are two or more weeks old concurrent with appearance of spines on dendrites of newly born neurons (Zhao et al., 2006). Four to six weeks after birth these neurons become fully integrated in the circuit (Jones et al., 2003). The stem cells that exist are a subset of astrocytes (Doetsch and Hen, 2005). In the SGZ, astrocyte-like stem cells divide to generate intermediate precursors, which remain in clusters of two to four cells in neurogenic niches that are formed by the processes of astrocytes and specialized vasculature (Seri et al., 2004). These cells progressively generate more differentiated progeny, which eventually mature into granule neurons. There are good markers for labeling different stages of the cellular progeny, but these markers usually label nuclei, soma, or somatodendritic compartments, but not axonal compartment. Nevertheless, if we adopt the theory of recapitulation or embryological parallelism to adult neurogenesis, it can be accepted that adult neurogenesis recapitulates neuronal development (Ming and Song, 2011). Thus, axonal cytoskeleton offers us some specific markers which participate in the process of morphological and physiological maturation that takes place in these cells after commitment to neuronal lineage. One of these proteins is tau protein.

## Tau Protein

Tau is a neuronal microtubule-associated protein that stabilizes neuronal microtubules under normal physiological conditions. Tau is able to promote polymerization of tubulins (Weingarten et al., 1975) and prevent their dynamic instability by its binding to microtubules (Drechsel et al., 1992). Tau plays a key role in the morphogenesis of neurons. The human *tau* gene contains 16 exons from which different tau isoforms are generated by alternative splicing (Goedert et al., 1989; Andreadis et al., 1992). Some of these isoforms are selectively expressed during embryonic and early postnatal development (Goedert et al., 1989, 1996; Lovestone and Reynolds, 1997). Exon 10 encodes one of the four repeat sequences (Goedert et al., 1989; Goedert and Spillantini, 2001) that form the microtubule-binding domain (Lee et al., 2001). The presence of exon 10 results in tau with four repeat microtubule-binding sequences (tau-4R), whereas the alternatively spliced isoforms without exon 10 have only three of these sequences (tau-3R). Isoforms lacking exon 10 are found at early developmental stages whereas tau isoforms containing exon 10 are mainly found in neurons at mature developmental stages (Avila et al., 2004). The microtubule-binding domain contains three or four similar but not identical repetitive sequences of 31 or 32 residues. Each of these repeats can be divided in two parts, one composed of an 18 residue sequence that contains the minimal region with tubulin binding capacity, and the second, a less conserved domain of 13 (or 14) residues known as the inter repeat. It should be noted that in the tubulin binding regions, the sequence with the highest capacity to bind to microtubules is that contained within the first repeat, the following inter region, and the second repeat (Goode et al., 1997). The repeats bind to microtubules and can promote microtubule assembly (Trinczek et al., 1995). As stated above, the alternative splicing of exon 10 may result in the expression of tau-3R or tau-4R in a cell, which in turn may produce some physiological differences in the cell. In fact, tau-4R binds microtubules with a greater affinity and can displace the previously bound tau-3R from microtubules (Lu and Kosik, 2001).

One of the main posttranslational modifications of tau protein is phosphorylation. Phosphorylation regulates the binding of tau to microtubules and to the membrane (Brandt et al., 1995). Phosphorylation appears to be the predominant way in which tau function can be regulated. Although different kinases may modify tau, there is emerging evidence that GSK3 plays an important role in regulating tau phosphorylation under normal (physiological) and pathological (tauopathies) conditions (Avila et al., 2004).

The phosphorylation of tau is developmentally regulated; it is higher in fetal neurons and decreases with age during the development (Brion et al., 1993). Interestingly, phosphorylation at different sites could take place in different tau isoforms (Hernandez et al., 2003). This could be due to the different cellular localization or subcellular compartmentalization of the different tau isoforms or the fact that different kinases/phosphatases can modulate tau phosphorylation in a different way.

## Tau-3R and Adult Neurogenesis

**Figure 1:**
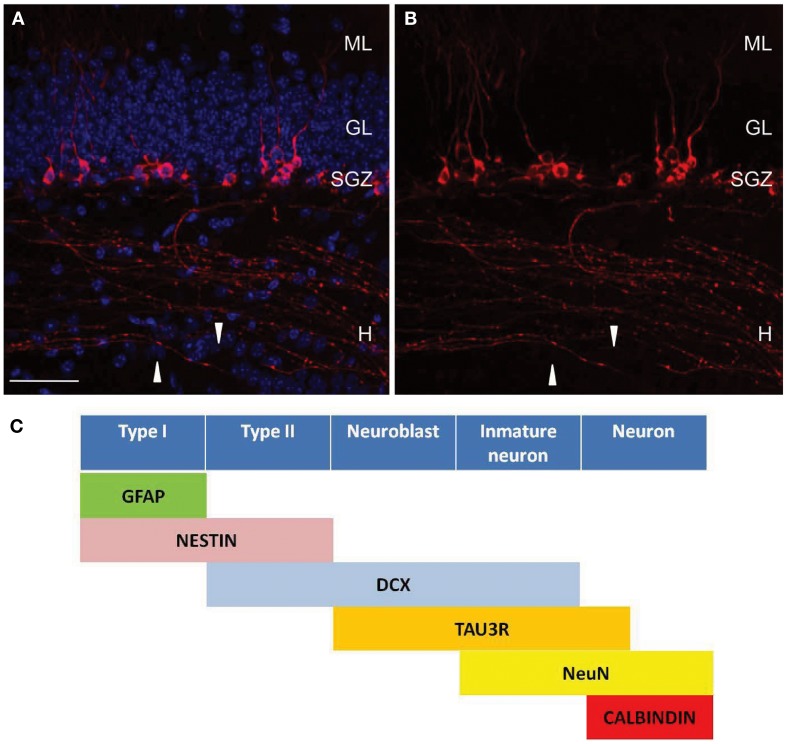
**Tau-3R expressing cells in the DG**. **(A,B)** Tau-3R (red) immunohistochemistry in 2-month-old wild-type (C57BL/6) mice showing the abundance of tau-3R expressing cells along the SGZ. White arrows indicate axonal processes. DAPI staining in blue. Scale bar: 50 μm. H, hilus; GL, granular layer; SGZ, subgranular layer. **(B)** Shows tau-3R immunolabeling. Tau-3R antibody labeled the somatic compartment of a subpopulation of cells in the SGZ of the hippocampal DG as well as axonal processes in the hilar region and hippocampal CA3 subfield. **(C)** Diagram indicating the lineage and marker expression during adult neurogenesis in SGZ including tau-3R as a new marker for axonal processes [**(A,B)** reprinted from Journal Alzheimer Disease (Llorens-Martin et al., 2011) with permission from IOS Press].

Adult newborn neurons express tau-3R (Figures 1A,B; Bullmann et al., 2007; Llorens-Martin et al., 2012). To study the temporal window or time course of tau-3R isoform expression in immature neurons BrdU-immunopositive cells colabeled for tau-3R demonstrated that tau-3R can be found 3 days after BrdU administration. The thymidine analog is observed reaching a maximum 14 days after BrdU incorporation (Bullmann et al., 2007). The same results were reached using retrovirus-mediated GFP transduction (Llorens-Martin et al., 2012). After stereotaxic injection of retrovirus, which only infects dividing cells, mice were killed at different time points and GFP and tau-3R expression were analyzed by immunohistochemistry. GFP and tau-3R colabeling can be detected in a period of 7–21 days after viral infection. Thus, both approaches demonstrate that there is a transient expression of tau-3R isoform during adult neurogenesis. Retrovirus injection allowed analyzing axogenesis. Thus, differentiated or long axonal processes were rarely observed 1 week after viral injection and axonal tau-3R immunolabeling was almost absent in these cells, but a slight somatodendritic staining was observed in some of the cells analyzed. Two-week-old neurons showed apical dendrites and axons in the hilar region. These axons, as well as the somatic compartment were labeled with tau-3R antibody. Three-week-old neurons had more elaborate dendritic arborization. In cells of this age, tau-3R was found in the somatic compartment of most of the cells, but only few of them expressed tau-3R in the axons. This tau-3R staining was detectable both in the subgranular layer where newborn axons begin, as well as in hilar region and CA3 field. Finally, 8-week-old neurons did not express neither axonal nor somatic tau-3R.

In the majority of the labeled cells DCX and tau-3R colocalize and only a few of them are single DCX-positive labeled (Llorens-Martin et al., 2012). These cells probably represent those cells which initiate a neuronal program but are in the initial stage of neuronal maturation. However, some cells labeled with anti-calbindin antibody contain tau-3R (Bullmann et al., 2007). Thus, some mature granule cells express the tau-3R protein. Although it is possible that the change in alternative splicing occurs in mature granule cells, a possible explanation could be that half-life of tau protein is high (about 60 h in HT22 cells) and higher into its phosphorylated form (Poppek et al., 2006).

By using an specific antibody, Bullmann et al. (2007) have also demonstrated that tau protein expressed in these newborn neurons lack of exon 2 and 3. These N-terminal exons regulate the binding of tau to the membrane (Brandt et al., 1995) and are expressed during embryonic and early postnatal development (Kosik et al., 1989).

## Tau Phosphorylation and Adult Neurogenesis

In addition to the presence or absence of exon 10, the phosphorylation of tau is developmentally regulated: it is higher in fetal neurons and decreases with age during development (Brion et al., 1993, 1994; Yu et al., 2009). However, fetal-tau phosphorylation can also be observed in the adult. It has been demonstrated that the presence of tau phosphorylated in fetal epitopes is related with adult neurogenesis in the SGZ, although fetal-tau phosphorylation can be found in adults not only in these areas (Yu et al., 2009). Tau phosphorylated is coexpressed temporally and spatially with DCX (Fuster-Matanzo et al., 2009; Hong et al., 2010) and neuroD (Hong et al., 2010) in the hippocampal dentate gyrus.

In the central nervous system GSK3 is the main tau kinase. GSK3 is inhibited by phosphorylation in the N-terminal end. Interestingly GSK3β phosphorylated in the inhibitory Ser-9 does not colocalize with DCX, suggesting that active GSK3β is the main tau kinase in newborn neurons (Hong et al., 2010).

As shown above, it is well known that phosphorylated tau has a reduced affinity for microtubules. In good agreement, it has been previously shown that tau hyperphosphorylation in transgenic mice overexpressing GSK3β correlates with somatodendritic accumulation of microtubule-unbound tau in hippocampal neurons (Lucas et al., 2001). The somatodendritic localization of phosphotau in adult newborn neurons from SGZ was evidenced by immunohistochemistry with the 7.51 antibody (Fuster-Matanzo et al., 2009) that recognizes the microtubule-binding domain of tau and that, accordingly, detects only unbound tau (Novak et al., 1991).

Although these studies have been mainly carried out in the hippocampus, the same seems to occur in the SVZ, the other main neurogenic area in adult rodents (Fuster-Matanzo et al., 2009; Hong et al., 2011).

## Human and Rodent Differences

Can these studies be translated to humans? First it has been demonstrated that in adult human brain neurogenesis takes place in the SGZ of the hippocampus while generation of new neurons from SVZ destined to the olfactory bulb is mainly limited to early childhood and decline in the adulthood (Guerrero-Cazares et al., 2011; Sanai et al., 2011; Wang et al., 2011). Recently, it has been demonstrated that ^14^C concentrations in genomic DNA correspond to the atmospheric levels at the time of birth of the individuals, establishing that there is very limited postnatal neurogenesis in the human olfactory bulb (Bergmann et al., 2012). Whereas in the human adult central nervous system, six different tau isoforms are expressed that differ in the presence or absence of exons 2, 3, and 10 (Goedert and Spillantini, 2001) in mice only tau-4R is expressed in adult neurons. Thus, tau-4R is not present in fetal human brain in contrast to adult human brain (Goedert et al., 1989; Kosik et al., 1989; Goedert and Jakes, 1990). The same has been observed in fetal mouse brain (Janke et al., 1999; Kampers et al., 1999). Interestingly some differences have been observed in adult brain in the expression of tau isoforms. Tau-3R isoforms are not present in mature neurons of adult rodents (Brion et al., 1993; Spillantini and Goedert, 1998) while the adult human brain contains both tau isoforms (Avila et al., 2004). Thus, although it is likely that in human adult dentate gyrus the newborn neurons in the SGZ also express fetal-tau, this has yet to be demonstrated.

## Tau Function in Adult Neurogenesis

New neurons require a high degree of plasticity to migrate, differentiate, send axons to CA3, and integrate in the granule cell layer. Tau-4R isoforms promote microtubule assembly at a faster rate than the tau-3R isoforms (Goedert and Jakes, 1990), suggesting that tau-3R protein may aid newborn neurons to differentiate by decreasing microtubule stability. This idea is supported by the expression of tau protein during brain development. To determine the role of tau protein in adult neurogenesis, that process has been studied in tau-KO mice. Thus, two tau-KO lines have been analyzed. One of those tau-KO lines (generated from a strain that express exclusively the human tau (Andorfer et al., 2003) and crossed with wild-type mice) shows a decrease in immunoreactivity of neuroD and DCX-positive neurons (Hong et al., 2010). However, in the other line (Dawson et al., 2001) number of DCX-positive cells was similar in tau-KO mice compared with wild-type (Fuster-Matanzo et al., 2009). Nevertheless, in the last model DCX-positive cells were uniformly aligned with the SGZ while some DCX cells in wild-type mice could be found in the granular cell layer. Quantification of DCX-positive migrating cells demonstrated that tau-KO mice show a decrease in migration compared with wild-type animals. Thus, although differences among both models are not actually clear, it certainly demonstrates that tau has a role in adult neurogenesis. These results suggest that tau protein facilitates DCX-positive cells migration. DCX-positive cells have to migrate from the SGZ to upper layers and send axons into the CA3 region. This process requires a dynamic microtubule network. Keeping this in mind, it is not strange to find phosphorylation of tau, as phosphorylation decreases the affinity of tau protein for microtubules. Interestingly, phosphorylated tau is accumulated in the somatic compartment, something that is also observed in developing neurons (Brion et al., 1994). These results suggest that phosphorylation of tau during neurogenesis gives DCX-positive cells a less stable and more dynamic microtubule network. The need for a dynamic cytoskeleton is also supported by the observation that DCX-positive cells expresses tau-3R isoform.

An increase in hippocampal neurogenesis has been observed in Alzheimer disease, the main tauopathy (Jin et al., 2004). However, the relationship among tau protein from adult newborn neurons and tauopathies is limited. Some data show that expression of tau protein with three mutations found in frontotemporal dementia with parkinsonism associated to chromosome 17 (FTDP-17) results in a decrease of the dentate gyrus ventral blade, apparently due to a reduction in the proliferation of neuronal precursors (Llorens-Martin et al., 2011). Also, triple transgenic mice harboring three mutant genes (beta-amyloid precursor protein, presenilin-1, and tau) have an impaired ability to generate new neurons in the DG of the hippocampus (Rodriguez et al., 2008). Nevertheless it should be remarked that in most of those neurodegenerative disorders, tau protein presents alterations in tau isoform splicing and is phosphorylated in the same epitopes found in adult neurogenesis (Buee et al., 2000).

## Tau-3R, A New Marker to Study Adult Neurogenesis

Fluorescent retrograde tracers (Hastings and Gould, 1999) and retroviral expression of green florescent protein have been used to visualize new axons (Toni et al., 2008). However, inflammation, degeneration, or DG structure alteration may be an important technical disadvantage that should be taken into account. We propose tau-3R as a new molecular marker to be used in adult neurogenesis (Figure 1C). As reviewed above, this tau isoform labels new axons from newborn granule cells which send axons into the CA3 region from SGZ. Tau protein isoform with three microtubule-binding domains is a marker of those axons following an antigen retrieval protocol (Llorens-Martin et al., 2012). The labeling of sagittal sections with tau-3R colocalizes with a subpopulation of DCX-positive cells. Several molecular markers have been used to analyze production of these new neurons, although no markers for new axons have been described. Thus, most of the actual investigations in adult neurogenesis only focus their studies in the somatodendritic compartment, while the axonal one has not been extensively analyzed because the lack of appropriate immunological tools.

Adult hippocampal neurogenesis is influenced by external stimuli, such as physical exercise, and by intrinsic conditions like age and disease. Thus, young exercised mice show higher number of tau-3R labeled immature axons in the granule cell layer of the mouse hippocampus (Llorens-Martin et al., 2012). Another model where a modulation of adult neurogenesis is observed is aging. During aging, a decrease in the number of immature neurons has been reported (Lazarov et al., 2010) and, in good agreement, a reduction in the number of immature axons labeled with tau-3R antibody can be observed (Llorens-Martin et al., 2012).

A common feature of several neurodegenerative diseases is the alteration of adult hippocampal neurogenesis rate (Lazarov et al., 2010). Thus, a murine model of FTDP-17, in which mutated human tau expression is known to be related to a dramatic reduction in the number of immature neuroblasts (Llorens-Martin et al., 2011), immature axons appear slightly disorganized in the hilar region and a reduced number of tau-3R positive axons can be appreciated in this region. Furthermore, aged AβPPind,sw mice with abundant hippocampal amyloid-β plaques cannot be detected neither immature neuroblasts nor immature axons (Llorens-Martin et al., 2012).

## Conclusion

To summary, recent studies favor the suggestion that tau-3R provides a dynamic microtubule network in DCX-positive cells which allow a proper axonal growing in adult neurogenesis. This role is facilitated by tau phosphorylation. From a practical point of view, tau-3R can be a new molecular marker to be used in adult neurogenesis. This tau isoform labels new axons from newborn granule cells. Taking into account that several molecular markers have been used to analyze production of these new neurons but no markers for new axons have been described, we conclude that labeling of sagittal sections with tau-3R would be an efficient marker and a valuable tool to study new axons in the SGZ. A “proof of principle” of the power of that tau-3R labeling has been demonstrated showing modulation of tau-3R positive axons under physiological conditions (exercise and aging) and diverse neurodegenerative (FTDP-17 and Alzheimer disease) models.

## Conflict of Interest Statement

The authors declare that the research was conducted in the absence of any commercial or financial relationships that could be construed as a potential conflict of interest.
